# Exploring School Students’ Knowledge and Expectations of Careers in Psychology, Psychiatry and Mental Health Nursing: A Thematic Analysis

**DOI:** 10.1192/bjo.2022.191

**Published:** 2022-06-20

**Authors:** Hannah Fosker, Hayley Andrews, Sarah Addison, Rachel Winter

**Affiliations:** 1 Leicestershire Partnership NHS Trust, Leicester, United Kingdom; 2 Leicester Medical School, University of Leicester, Leicester, United Kingdom

## Abstract

**Aims:**

Attracting more doctors and nurses to mental health careers is vital to support the growing demand for mental health services. Despite low numbers of doctors choosing psychiatry, and a shortage of mental health nurses, psychology degrees remain a popular choice. This study explores the understanding and knowledge students studying psychology A Level have about mental health careers, and the careers guidance they have received. We ask ‘are students who are interested in studying psychology at university an untapped resource for recruitment to psychiatry and mental health nursing?’.

**Methods:**

Focus groups were held with A-Level psychology students considering applying to university to study psychology. Focus group discussions were recorded, transcribed and anonymised and were analysed using thematic analysis.

**Results:**

Three key themes were identified. Firstly, student interest in psychology as a degree subject (with mental illness, neurobiology and human behaviour cited as key interests). Secondly, student motivation for a future career in which they would have a therapeutic role working with people with mental illnesses. Thirdly, student knowledge, or lack of it, around what a career in psychology or other mental health careers would entail, and the pathways to these.

**Conclusion:**

There remains uncertainty in young people regarding what the different types of mental health practitioner roles are, and the career pathways to these. More detailed, accurate information made available to students interested in working with people with a mental illness may lead to more students considering a career in mental health nursing or medicine (and then psychiatry) as an alternative to a psychology. It is important that those aspiring to become clinical psychologists understand the qualifications required and competitive nature of this profession. Inaccurate information runs the risk of students acquiring significant university debt, only to find they are not qualified for the type of role they envisaged. A lack of accurate, high quality and timely careers information may also be depriving psychiatry and mental health nursing of enthusiastic, able and motivated students. This study adds support to the need for better careers guidance and awareness around mental health careers for school and sixth form students.

